# Delayed Hospital Discharge–The Effect of Dementia and GP Contacts Prior to Hospitalization

**DOI:** 10.1093/geroni/igaa057.444

**Published:** 2020-12-16

**Authors:** Mari Aaltonen, Shiraz El Adam, Mariko Sakamoto, Anne Martin-Matthews, Kimberlyn McGrail

**Affiliations:** 1 Tampere University, Tampere, Finland; 2 The University of British Columbia, Vancouver, British Columbia, Canada; 3 University of British Columbia, Vancouver, British Columbia, Canada

## Abstract

Delayed hospital discharge, also known as alternate level of care (ALC) in Canada, refers to a stay in hospital when acute services are no longer needed but the patient occupies a hospital bed while waiting to be discharged to an appropriate care setting. ALC has negative consequences for both the service system (high costs, inappropriate use of hospital resources) and individuals (feelings of uncertainty, functional loss). Extensive administrative health care data from British Columbia, Canada, were employed to study differences in ALC between people with and without dementia in 2001/02, 2005/06, 2010/11 and 2015/16, and whether continuity in physician–patient relationships prior to hospitalization is associated with ALC. We analyzed designation of ALC and length of stay in ALC, using generalized estimating equations logistic regression and negative binomial regression analysis. Of all individual, residential area and health care related factors, dementia was the single most important factor increasing the odds of designation of ALC (OR 4.76, 95%CI 4.59, 4.93). Dementia also added to the length of stay in ALC. Proportion of patients designated to ALC increased over the study years. Higher number of visits to the same general practitioner (GP) prior to hospitalization decreased the odds of ALC, especially in people with dementia. As populations age, the number of people with dementia is increasing. Efforts to control ALC have resulted in greater concentration of ALC among people with dementia. Higher continuity GP care may be a way to help understand and control these trends.

